# *Helicobacter pylori* Protein JHP0290 Exhibits Proliferative and Anti-Apoptotic Effects in Gastric Epithelial Cells

**DOI:** 10.1371/journal.pone.0124407

**Published:** 2015-04-16

**Authors:** Raquel Tavares, Sushil Kumar Pathak

**Affiliations:** Department of Molecular Biosciences, The Wenner-Gren Institute, Stockholm University, Stockholm, Sweden; Institut Pasteur Paris, FRANCE

## Abstract

The influence of *Helicobacter pylori* infection on gastric epithelial cell proliferation, apoptosis and signaling pathways contributes to the development of infection-associated diseases. Here we report that JHP0290, which is a poorly functionally characterized protein from *H*. *pylori*, regulates multiple responses in human gastric epithelial cells. The differential expression and release of JHP0290 homologues was observed among *H*. *pylori* strains. JHP0290 existed in monomeric and dimeric forms in *H*. *pylori* cell extracts and culture broth. Recombinant purified JHP0290 (rJHP0290) also showed monomeric and dimeric forms, whereas the rJHP0290 C162A mutant exhibited only a monomeric form. The dimeric form of the protein was found to bind more efficiently to gastric epithelial cells than the monomeric form. The exposure of gastric epithelial cells to rJHP0290 induced proliferation in a dose-dependent manner. Faster progression into the cell cycle was observed in rJHP0290-challenged gastric epithelial cells. Furthermore, we detected an anti-apoptotic effect of rJHP0290 in gastric epithelial cells when the cells were treated with rJHP0290 in combination with Camptothecin (CPT), which is an inducer of apoptosis. CPT-induced caspase 3 activation was significantly reduced in the presence of rJHP0290. In addition, the activation of ERK MAPK and the transcription factor NFκB was observed in rJHP0290-challenged gastric epithelial cells lines. Our results suggest that JHP0290 may affect *H*. *pylori*-induced gastric diseases via the regulation of gastric epithelial cell proliferation and anti-apoptotic pathways.

## Introduction

*Helicobacter pylori* is a helix-shaped, Gram-negative bacterial pathogen that colonizes the gastric mucosa of more than half of the human population worldwide [1]. Although infection with this bacterium is primarily asymptomatic, persistence may lead to severe gastroduodenal pathologies, such as chronic gastritis, peptic ulcers, gastric adenocarcinoma and mucosa-associated lymphoid tissue (MALT) lymphoma [2]. The inflammatory host responses to *H*. *pylori* colonization of the gastric mucosa are largely ineffective with respect to eliminating the bacterium. Consequently, infected individuals become susceptible to mucosal damage [3] and bacterial survival in the acidic environment of the human stomach is favored.

The maintenance of gastric epithelial cell homeostasis is essential for the normal function of the gastrointestinal mucosa. Infection with *H*. *pylori* is associated with a disruption of the equilibrium between cell growth and cell death; this disruption contributes to the development of *H*. *pylori* infection-associated diseases [4–8]. Several studies provided evidence supporting the influence of *H*. *pylori* and bacterium-derived products on gastric epithelial cell proliferation not only in gastric cancer cell lines but also in gastric biopsies [4, 6, 8–17]. *H*. *pylori* infection may also lead to the induction of anti-apoptotic signaling pathways and the expression of anti-apoptotic genes, such as induced myeloid leukemia cell differentiation protein (Mcl-1) and cellular inhibitor of apoptosis protein 2 (cIAP-2), in gastric epithelial cells [18–23]. In contrast, other studies reported that the bacterium is equally capable of inducing apoptosis in gastric epithelial cells [10, 11, 24–27]. The major virulence factor cytotoxin-associated gene A (CagA) regulates epithelial cell proliferation and anti-apoptotic pathways in both the phosphorylated and non-phosphorylated forms [19, 28]. The co-expression of CagA and *H*. *pylori* heat shock protein B (HspB) was found to induce gastric epithelial cell proliferation independent of bacterial infection [29]. *H*. *pylori* lipopolysaccharide (LPS) and the SlyD protein have also been demonstrated to induce proliferation and anti-apoptotic signaling pathways in gastric epithelial cell lines [15, 23]. *H*. *pylori* infection alters cell cycle progression in gastric epithelial cells. Arrest at G1 phase has been reported in several studies. However, the effect appears to be dependent on the bacterial strain, the cell line and the multiplicity of infection (MOI) of the bacterium. Ding et al. reported that when the MOI was greater than or equal to 150:1, the cell cycle was arrested at the G1 phase. However, at lower MOIs, the cell cycle was not arrested at G1 and progression into S phase was observed, indicating that the regulation of the cell cycle is complex [30]. Peek et al. demonstrated that *H*. *pylori* strain-specific factors regulate the faster progression of the cell cycle from G1 into G2-M in AGS cells after 6 h [16]. The co-expression of CagA and HspB induces a faster progression into the cell cycle in gastric epithelial cells [29].

*H*. *pylori* infection leads to the activation of multiple signaling pathways, including ERK MAPK and the transcription factor NF-κB, in gastric epithelial cells. ERK MAPK is involved in the regulation of inflammatory responses, apoptosis, proliferation and the cell cycle in *H*. *pylori-*infected gastric epithelial cells [30–33]. NF-κB is a key regulator of the immune response against *H*. *pylori* infection and is known to modulate genes involved in the control of inflammation, cell proliferation and apoptosis [22, 34–36]. Among *H*. *pylori* virulence determinants, secreted proteins are believed to play important roles in bacterial adaptation to the mucosal environment and in the regulation of host cell responses due to the generally non-invasive nature of the bacterium. Our work has focused on the secreted protein HP0305, which is overexpressed in *H*. *pylori* under acidic stress [37, 38]. HP0305 is strongly recognized by sera from *H*. *pylori-*infected patients and was identified as a potential biomarker for gastric cancer risk in China [39, 40]. Seropositivity to HP0305 is associated with a significant 60–80% increase in colorectal cancer risk [41]. Our previous study indicated that JHP0290, which is a homolog of HP0305, binds to various cell types and regulates macrophage responses [42].

In this report, we studied the expression and release of JHP0290 homologues in multiple strains of *H*. *pylori*. Further, to understand the role of JHP0290 in *H*. *pylori* pathogenesis, we investigated the responses of gastric epithelial cells to the purified protein. We provide evidence supporting the differential expression of the protein homologues among *H*. *pylori* strains. rJHP0290 was found to induce proliferation in gastric epithelial cells. Furthermore, rJHP0290 was observed to exert an anti-apoptotic effect when the cells were treated with this factor in combination with an apoptosis inducer. In addition, rJHP0290 was found to activate ERK MAPK and NF-κB in gastric epithelial cells. These results provide mechanistic insight into the role of JHP0290 in *H*. *pylori* pathogenesis.

## Materials and Methods

### Bacterial strains and culture conditions

*H*. *pylori* strains J99 (ATCC 700392), 26695 (ATCC 700392D-5), HPAG1 and the *jhp0290*-deficient mutant strain J99 *Δjhp0290* have been described previously [42, 43]. The TN2GF4, P12, and CCUG17875 strains were kindly provided by Anna Arnqvist from Umeå University. The bacteria were grown at 37°C under microaerophilic conditions on Columbia blood agar plates (Acumedia) supplemented with 8% horse blood and 8% horse serum. For liquid cultures, the cells were grown in Brucella broth (Acumedia) containing 8% horse serum at 37°C under microaerophilic conditions with shaking. The *Escherichia coli* BL21 (DE3) and *E*. *coli* DH5α strains were grown on Luria-Bertani Miller (LBM) medium.

### Cell culture and treatments

The AGS (CRL-1739) and MKN45 (JCRB0254) human gastric epithelial cell lines were obtained from the American Type Culture Collection and the Japan Health Science Research Resource Bank, respectively. The cells were cultured in RPMI-1640 (Invitrogen) supplemented with 10% heat-inactivated fetal calf serum (FCS) (Invitrogen) at 37°C in a humidified 5% CO_2_ atmosphere. Human primary stomach epithelial cells (HPSEC) were obtained from CellBiologics, USA. HPSEC were cultured in epithelial cell medium (M6621, CellBiologics) following the instructions from the provider. As indicated, the cells were treated with purified rJHP0290 or the same volume of protein storage buffer.

### *H*. *pylori* protein extraction and immunoblotting

The protocols used for cell lysate preparation and immunoblotting were described previously [42]. Briefly, 1x10^8^ bacteria were mixed with 100 μl of SDS-PAGE sample buffer (45 mM Tris-Cl, pH 6.8, 10% glycerol, and 1% SDS in the presence or absence of 5% β-mercaptoethanol or 50 mM DTT). A 10 μl sample of cell lysate or 20–40 μl of filtered culture supernatant was separated on a 12.5% polyacrylamide gel under reducing or non-reducing conditions and transferred electrophoretically to polyvinylidene difluoride membranes (Millipore). The blots were blocked with 5% (w/v) non-fat dry milk (NFDM) in PBS containing 0.1% (v/v) Tween 20 (PBST) for 1 h and subsequently incubated overnight at 4°C with an anti-JHP0290 or anti- alkyl hydroperoxide reductase (AhpC) or anti-Urease α (bC-14) (Santa Cruz Biotechnology, SC-22445) antibody in blocking buffer. Protein A affinity-purified polyclonal antibody against JHP0290 and AhpC was generated by EZbiolab (USA). After washing with PBST, the membranes were incubated with a secondary antibody conjugated to the Odyssey IR-dye (Li-COR) for 1 h. The membranes were visualized using an Odyssey IR scanner (Li-COR). The band intensities of the immunoblots were quantified using the ImageJ software (NIH, Bethesda, MA, USA).

### Cloning, expression and purification of wild-type (Wt) rJHP0290 and rJHP0290 C162A

Wt rJHP0290 (18–184 aa) was purified as described previously [42]. To generate a construct for the expression of rJHP0290 C162A, site-directed mutagenesis was performed using overlap extension PCR. The employed primers are depicted in Table 1. The initial rounds of PCR were performed using primer pair a and c for one reaction, primer pair b and d for the second reaction and *jhp0290* in pET28b^+^ as the template. The products of each PCR were gel-purified and used as templates for the second round of PCR with primers a and d. The final products were cloned between the NdeI and XhoI sites of the vector pET28b^+^. The constructs were verified by sequencing. rJHP0290 C162A was overexpressed and purified under conditions similar to those described for rJHP0290 Wt [42]. Briefly, the plasmid constructs were transformed into *E*. *coli* BL21 (DE3). The induction of the protein was performed at 37°C for 2–3 h with isopropyl thio-β-d-galactoside (100 μM). Hexa-His-tagged proteins were purified from the soluble fraction of the lysates via chromatography on Talon resin (Clontech). The purified protein was dialyzed extensively overnight to remove imidazole, and after fractionation on a 12.5% SDS-polyacrylamide gel and staining with the Coomasie brilliant blue dye, the proteins appeared as a single band under reducing conditions. To detect the monomeric and dimeric forms of rJHP0290, the samples were separated in the presence or absence of 50 mM DTT. The dialyzed protein was further incubated with an endotoxin removal column (Pierce), and the endotoxin content of the purified protein was measured using an endotoxin detection kit (Pierce) with a detection limit of 0.1 EU/ml.

**Table 1:** Primers used in this study.

Name	Sequence (5’-3’)
Primer a	5’—TACGTAACATATGGTGGAATTTGGATCTATCT -3
Primer b	5’—GGCTAAACGAGCCGAGAGCTTTC—3’
Primer c	5’—GAAAGCTCTCGGCTCGTTTAGCC -3’
Primer d	5’—TTACTCGAGTTATCCCTTGATCATGCTT -3’

### Binding assay for rJHP0290 using FACS

Binding assays were performed as described previously [42]. Briefly, the cells were treated with Wt or mutant rJHP0290 (2 μg/ml) for 15 min, followed by extensive washing with PBS to remove unbound protein. The cells were incubated with an anti-JHP0290 antibody (1:5,000 dilution) in FACS buffer (2% BSA in PBS) for 1 h on ice, followed by washing with FACS buffer and incubation with an Alexa 488-conjugated anti-rabbit IgG antibody (Molecular Probes) (1:5,000 dilution) in FACS buffer for 30 min on ice. After incubation, the cells were washed twice with FACS buffer and analyzed using an LSRFortessa flow cytometer. FlowJo software (Tree Star, USA) was used for the data analysis.

### 3-(4,5-Dimethylthiazol-2-yl)-2,5-diphenyltetrazoliumbromide (MTT) cell proliferation assay

1 x 10^4^ cells were plated in each well of 96-well plates and incubated overnight in a 5% CO_2_ incubator. The following day, the cells were treated with the indicated concentrations of rJHP0290 or protein storage buffer. After the desired period of incubation, the cells were washed twice with RPMI-1640 medium without FCS. MTT solution (0.48 mM in RPMI-1640 medium without FCS) was added to the cells, and the plates were further incubated for 4 h at 37°C. The supernatant was carefully removed, and 50 μl of DMSO (Sigma-Aldrich) was added to each well to dissolve formazan crystals. The cells were incubated for 10 min at 37°C. Absorbance was measured using a microplate reader (POLARstar Omega, BMG Biotech) at a wavelength of 540 nm. The ratio of the absorbance of protein-treated cells relative to that of control cells was calculated and presented as a percentage of cell proliferation.

### BrdU cell proliferation assay

Cell proliferation was measured using a BrdU ELISA kit (QIA58, Calbiochem, San Diego, CA, USA) according to the manufacturer’s instructions. Briefly, 5 x 10^4^ cells were plated in each well of 96-well plates and incubated overnight in a 5% CO_2_ incubator. The cells were treated as indicated in the figure legends. The BrdU label was added 16 h prior to the termination of the assay. BrdU incorporation into the cellular DNA was measured using a microplate reader (Polarstar Omega, BMG Biotech). The BrdU proliferation assays were repeated four times, and each sample was measured in at least triplicate.

### Cell cycle analysis using flow cytometry

After treatment, the cells were detached by mild trypsinization and collected by centrifugation at 1,000 x g for 5 min at 4°C. The cells were washed with PBS, fixed in ice-cold 70% ethanol at 4°C and stained with 40 μg/ml of propidium iodide (PI, Sigma-Aldrich) and 40 μg/ml of RNAse A (Qiagen) in PBS for 30 min at 37°C. The DNA profile was generated via flow cytometry using an LSRFortessa flow cytometer (BD Biosciences). The FACS data were analyzed using FlowJo software. The cell cycle experiments were performed six times in at least duplicate.

### Measurement of apoptosis

The cells were treated with rJHP0290 (100 ng/ml) and/or CPT (10 μM) (Sigma-Aldrich), as indicated in the figure legends. The cells were stained with a FITC-conjugated Annexin V antibody (BD Biosciences) and PI (BD Biosciences) according to the manufacturer’s instructions. Briefly, 1x10^5^ cells in 100 μl of Annexin binding buffer (10 mM HEPES, pH 7.4, 140 mM NaCl, and 2.5 mM CaCl_2_) were mixed with 1 μl of FITC-conjugated Annexin V antibody. The mixture was incubated for 15 min at room temperature in the dark. PI (2.5 μl) was added to the cell suspension immediately prior to analysis. The relative number of Annexin V-positive and/or PI-positive cells was determined using flow cytometry. FlowJo software was used for the data analysis.

### Caspase 3 activation assay

The activity of caspase-3 was measured using the Caspase 3/7 activity assay kit (AAT Bioquest, Inc., USA) according to the manufacturer’s instruction. Briefly, 5 x 10^4^ cells were plated in each well of 96-well plates and incubated overnight in a 5% CO_2_ incubator. The cells were treated with rJHP0290 (100 ng/ml) and/or CPT (10 μM), as indicated, for 16 h, followed by the addition of 100 μl/well of caspase 3 assay solution. The plate was incubated at room temperature for 1 h, protected from light. The fluorescence intensity was measured at Ex/Em = 350/450 nm using a POLARstar Omega Instrument (BMG Labtech).

### NF-κB activation assay

AGS and MKN45 cells were transfected with the pNiFty-SEAP NF-κB-inducible reporter plasmid construct (InvivoGen) using Lipofectamine 2000 (Invitrogen) transfection reagent following the manufacturer’s instructions. 54 h after transfection, cells were treated with the indicated concentrations of rJHP0290 or protein storage buffer for 20 h. Lipoteichoic acid (LTA, 100 ng/ml, InvivoGen) was used as positive control. SEAP activity in the culture supernatant was detected using the QUANTI-Blue detection system (InvivoGen) following the manufacturer’s instructions.

### Preparation of mammalian cell lysates and immunoblotting

After the indicated treatments, the cells were washed three times with PBS. The cell lysates were prepared via the addition of SDS-PAGE sample reducing buffer, followed by heating at 95°C for 10 min. Immunoblotting was performed using anti-phospho ERK1/2 (D13.14.4E) (1:1,000 dilution), anti-ERK1/2 (137F5) (1:1,000 dilution) and anti-IκBα alpha (L35A5) (1:2,000 dilution) antibodies from Cell Signaling Technology. An anti-actin antibody (Millipore) (1:5,000 dilution) was used to confirm equal loading. The secondary antibodies included goat anti-rabbit IgG and goat anti-mouse IgG conjugated to IRdye800CW (Li-COR) (1:10,000 dilution). ImageJ software was used to quantify band intensities, and actin was used to normalize the total amount of protein loaded in each well.

### Statistical analysis

For statistical analysis, experiments were performed at least three times, each with ≤3 samples. The GraphPad Prism 6.0 software package was used for statistical analysis. Differences between multiple groups were analysed using one-way ANOVA. Student’s t-test was used to analyse difference between two groups. A *p* value of <0.05 was considered statistically significant.

## Results

### Expression of JHP0290 homologues in various strains of *H*. *pylori*

**Fig 1:**
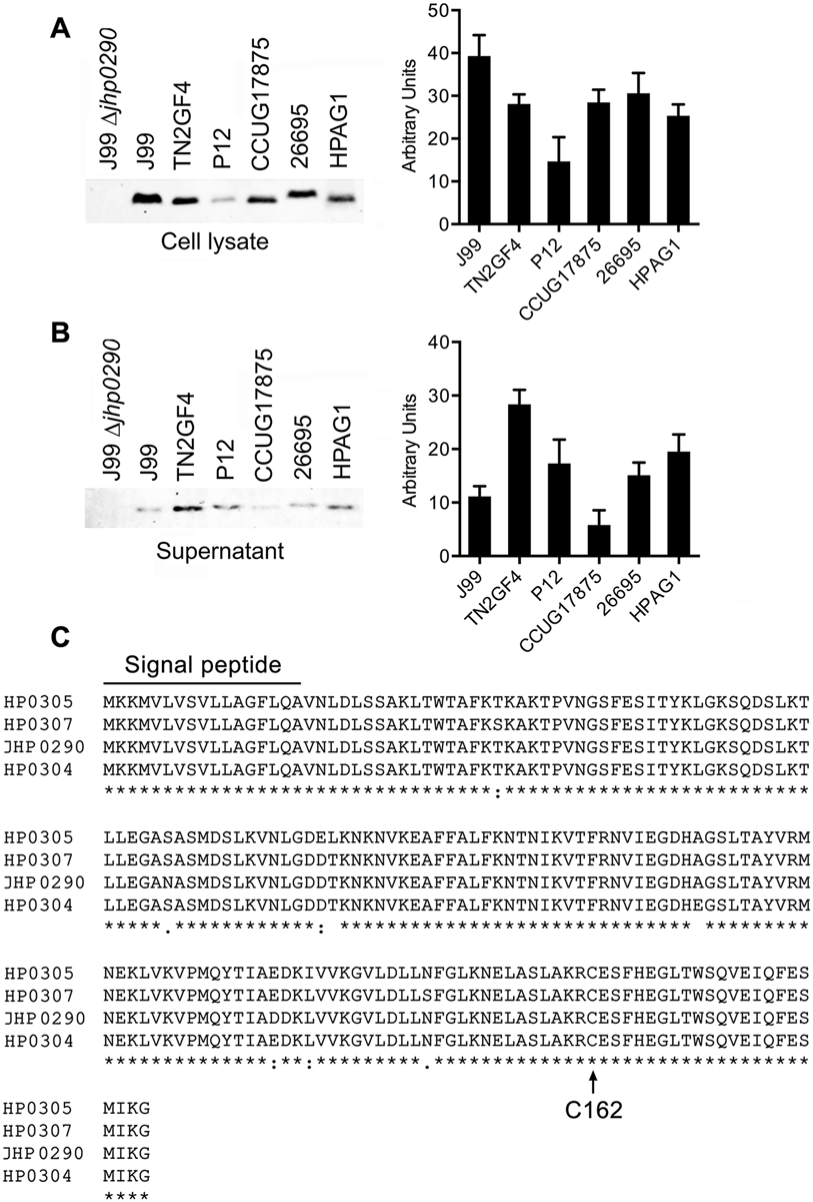
Expression of JHP0290 homologues in different *H*. *pylori* strains. Whole cell lysate **(A)** and culture broth (Supernatant) from equal number of cells **(B)** of various *H*. *pylori* strains as indicated in figure legends were immunoblotted with anti-JHP0290 antibody. Blot shown is representative of results obtained in more than five independent experiments. The graph shows western blot band intensities quantified by the ImageJ software. **(C)** Sequence homology analysis of JHP0290 homologues in *H*. *pylori* strains J99 (JHP0290), 26695 (HP0305), P12 (HPP12_HP0304) and HPAG1 (HPAG1_HP0307) using Clustal Omega software (EMBL-EBI). N-terminal 1–17 amino acids were identified as signal peptide by the SignalP 4.1 software (ExpPASy Bioinformatics Resource Portal). Conserved Cysteine at position 162 is marked by an arrow.

Sequence homology analysis indicated that JHP0290 is highly conserved among *H*. *pylori* strains. We first determined the expression of JHP0290 homologues in the whole cell extracts of several strains using the antibody raised against rJHP0290. Western blot analysis revealed that the protein was expressed by all of the strains that were tested in this study, although the expression level varied in different strains (Fig 1A). The same lysates were immunoblotted with antibodies against Urease and AhpC, which are abundantly expressed in *H*. *pylori*. The expression level of either Urease (data not shown) or AhpC (S1 Fig) did not vary significantly in the tested strains. The presence of a signal peptide (1–17 aa) in the sequence suggests that the protein is released by the bacterium. In a previous study, we reported that the JHP0290 protein of *H*. *pylori* strain J99 is released into the culture broth by the bacterium [42]. Kim et al. reported the secretion of a JHP0290 homolog (HP0305) by another *H*. *pylori* strain (NCTC 11637) [37]. To confirm the release of JHP0290 homologues by other strains of *H*. *pylori*, culture supernatants from various strains were immunoblotted with the anti-JHP0290 antibody. The release of JHP0290 homologues into the culture broth was observed for all strains (Fig 1B). Interestingly, the level of JHP0290 homologues released into the culture broth varied among strains; release did not correlate with the observed differences in expression levels. As shown in Fig 1A, the expression of the JHP0290 homologue in *H*. *pylori* strain TN2GF4 (Fig 1A, lane 3) was lower than that of the strain J99 (Fig 1A, lane 2); however, the level of protein released into the culture broth by TN2GF4 (Fig 1B, lane 3) was higher than that released by J99 (Fig 1B, lane 2). The same pattern was observed for strains P12 and HPAG1. The precise relationship between the amount of released JHP0290 protein homologues and the virulence of the bacterium requires further investigation.

### Monomeric and dimeric forms of rJHP0290 and binding to gastric epithelial cells

**Fig 2:**
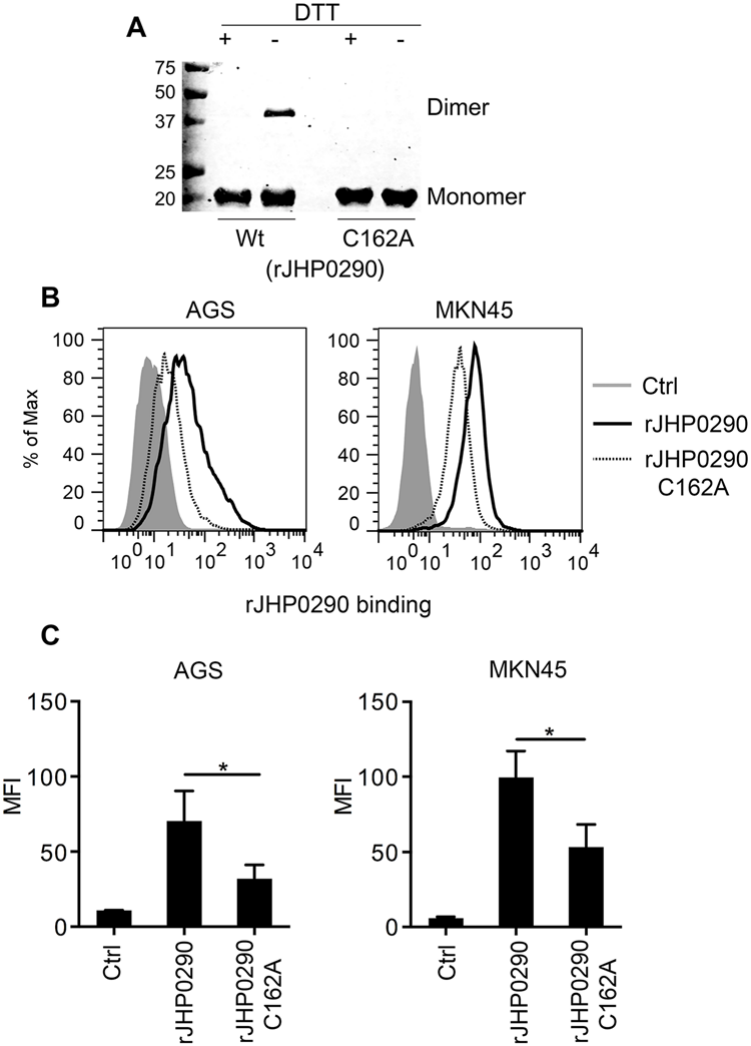
Binding of Monomeric and dimeric forms of rJHP0290 to AGS and MKN45 cells. **(A)** rJHP0290 Wt and rJHP0290 C162A in presence (+) or absence (-) of DTT in the SDS-PAGE sample buffer were immunoblotted with anti-JHP0290 antibody. Blot shown is the representative of results obtained in four independent experiments. **(B)** AGS and MKN45 cells were treated either with protein storage buffer (Ctrl) or with rJHP0290 Wt (2 μg/ml) or rJHP0290 C162A (2 μg/ml) for 15 min. Cells were washed, stained with anti-JHP0290 antibody and Alexa Fluor 488 conjugated secondary antibody followed by flow cytometry analysis. Result shown is the representative of results obtained in three independent experiments. **(C)** Flow cytometry results of the binding assay are depicted as the Mean Fluorescence intensity (MFI). Statistically significant differences are indicated by * (p < 0.05).

SDS-PAGE analysis of rJHP0290 revealed a single band of approximately 20 kDa in size (S2A Fig). However, under non-reducing conditions, another band of approximately 40 kDa in size was observed, suggesting that rJHP0290 was able to form a homodimer (S2A Fig). Immunoblotting with an anti-JHP0290 antibody further confirmed that the 20 kDa and 40 kDa bands were two forms of the same protein (Fig 2A). Because JHP0290 has a conserved cysteine at position 162 (Fig 1C), we assumed that the cysteine contributes to homodimer formation by rJHP0290 via disulfide bonds. To investigate the importance of the cysteine at position 162, we generated a mutant of rJHP0290 in which the Cysteine residue was mutated (rJHP0290 C162A). As expected, the mutant exhibited only a 20 kDa form under non-reducing conditions (Fig 2A and S2A Fig). Both the monomeric and dimeric forms of the JHP0290 protein were detected in culture broth and cellular extracts from *H*. *pylori* J99, indicating that both forms occurred naturally in the bacterium (S2B Fig). JHP0290 existed primarily in a monomeric form both when purified (rJHP0290) and in bacterial lysates or culture supernatants.

Because we previously observed the binding of rJHP0290 to various cell types [42], we further compared the ability of the monomeric and dimeric forms of the purified protein to bind to gastric epithelial cell lines. AGS and MKN45 cells were incubated with the same concentration of either rJHP0290 Wt or rJHP0290 C162A, followed by washing of unbound protein and staining of the cells with an anti-JHP0290 antibody. The binding of the Wt rJHP0290 was significantly higher than that of the rJHP0290 C162A (Fig 2B and 2C). Collectively, these results indicated that JHP0290 exits in two forms (i.e., the monomer and the dimer) and that the dimeric form of rJHP0290 binds more efficiently to gastric epithelial cells than the monomeric form.

### rJHP0290 induces proliferation in gastric epithelial cells

**Fig 3:**
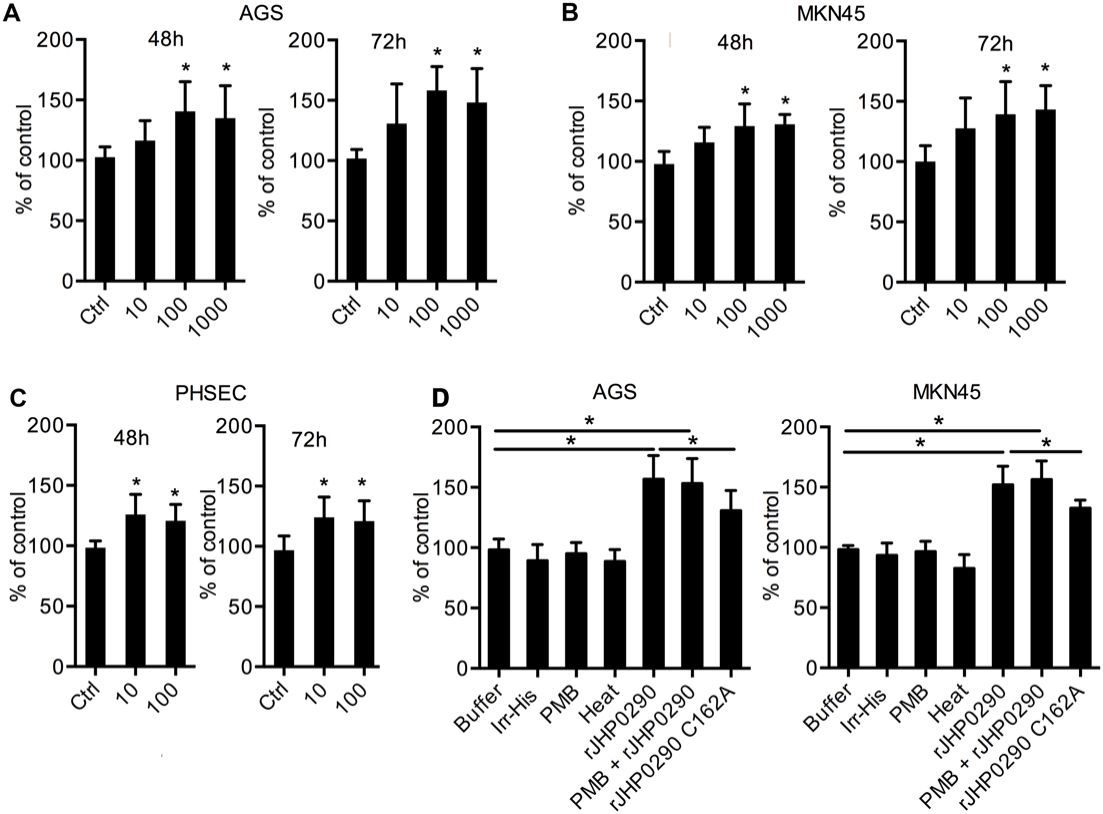
Effect of rJHP0290 on gastric epithelial cell proliferation. AGS **(A)**, MKN45 **(B)** and PHSEC **(C)** cells were incubated either with protein storage buffer (Ctrl) or different concentrations of rJHP0290 as indicated in the figure legends for 48 h and 72 h, followed by MTT assay to assess proliferation. Values indicate mean ± SD of six independent experiments performed at least in triplicate. **(D)** AGS and MKN45 cells were treated for 48h with buffer (ctrl) or rJHP0290 Wt (100 ng/ml) or rJHP0290 C162A (100 ng/ml) or an irrelevant His-tagged protein (Irr-His, 100 ng/ml). rJHP0290 was subjected to boiling for 30 min (heat) or was treated with polymyxin B (PMB) for 1 h before treatment of cells. The relative BrdU incorporation in cells was measured. Values indicate mean ± SD of four independent experiments performed in triplicate. Statistically significant differences are indicated by * (p < 0.05).

*H*. *pylori* infection is associated with the enhanced proliferation of gastric epithelial cells, and several virulence factors that regulate proliferation have been identified [15, 23, 29]. Considering the ability of rJHP0290 to bind to epithelial cells, we further studied the effect of rJHP0290 on gastric epithelial cell proliferation. AGS, MKN45 and PHSEC cells were treated with various concentrations of rJHP0290 for various periods of time. An MTT assay revealed that rJHP0290 could induce gastric epithelial cell proliferation in a dose-dependent manner (Fig 3A–3C). A BrdU cell proliferation assay, which is an additional method for assessing cell proliferation, further confirmed the proliferation-promoting property of rJHP0290 (Fig 3D). To rule out the possibility that the His-Tag contributes to the observed effect, an irrelevant His-tagged protein (Irr-His) was used to treat both cell lines under similar conditions. Irr-His failed to induce proliferation in both cell lines (Fig 3D), suggesting that the His-tag was not involved. Heat treatment of rJHP0290 inhibited the proliferation-inducing ability of rJHP0290, supporting the view that proliferation induction occurred due to the protein rJHP0290 (Fig 3D). Further, treatment with the LPS antagonist Polymyxin B (PMB) had no effect on rJHP0290-induced proliferation (Fig 3D), ruling out the possibility that the observed induction of proliferation was due to LPS contamination. The rJHP0290 C162A mutant was significantly impaired in its ability to induce gastric epithelial cell proliferation, which was expected due to the poor binding of the mutant to gastric epithelial cells (Fig 3D).

**Fig 4:**
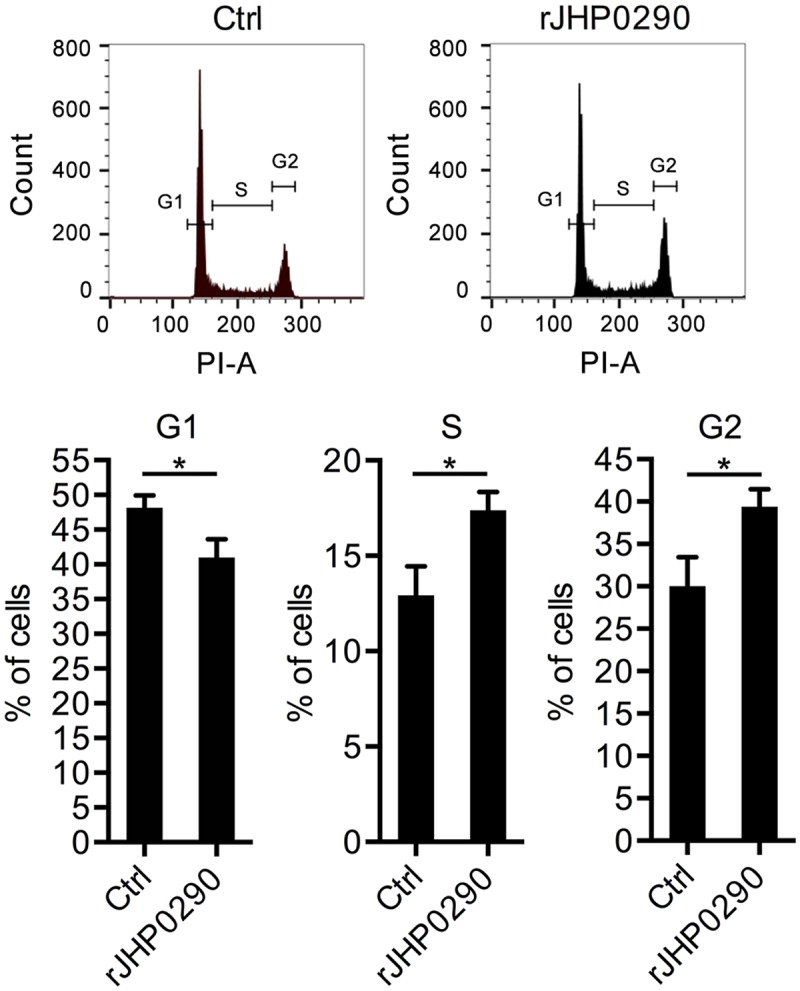
rJHP0290 modulates cell cycle progression in gastric epithelial cells. AGS cells were incubated either with protein storage buffer (Ctrl) or rJHP0290 (100 ng/ml) for 24 h. Cells were washed with PBS, fixed in ice-cold 70% ethanol at 4°C and stained with PI and RNAse A in PBS for 30 min. The DNA profile was generated by FACS. One representative profile (A) and mean ± SD of six independent experiments (B) are shown. Statistically significant differences are indicated by * (p < 0.05).

We further analyzed the effect of rJHP0290 on the gastric epithelial cell cycle. A representative cell cycle analysis is shown in Fig 4A. The incubation of AGS cells with rJHP0290 resulted in a faster G1/S phase transition. Significantly larger numbers of cells were observed in S-phase and G2-M phase in the rJHP0290-treated groups (Fig 4B). The above results indicated that rJHP0290 induces a faster progression into the cell cycle, resulting in increased proliferation.

### Resistance to apoptosis in rJHP0290-challenged AGS cells

**Fig 5:**
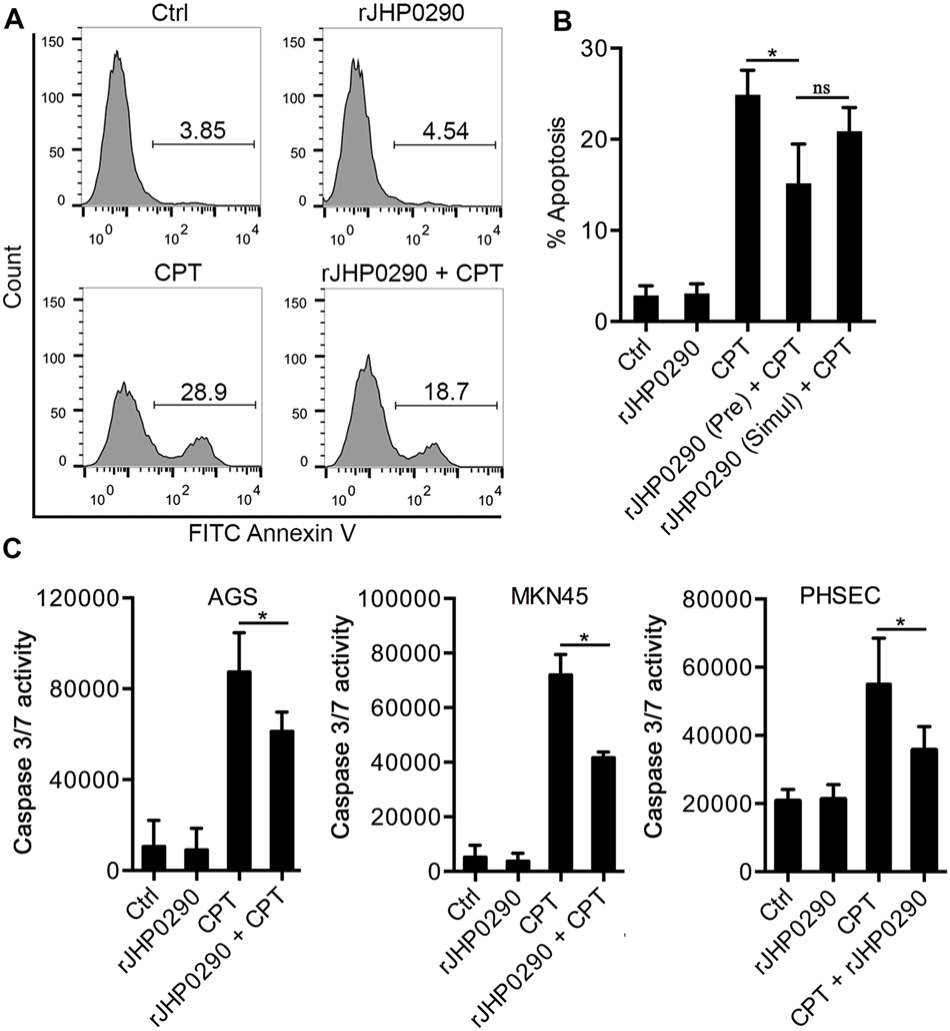
Anti-apoptotic effect of rJHP0290 on epithelial cells. **(A)** AGS cells were incubated either with protein storage buffer (Ctrl) or rJHP0290 (100 ng/ml) for 2 h followed by addition of corresponding volume of vehicle control or Camptothecin (CPT, 10 μM) for 20 h. Cells were washed, stained with FITC-conjugated Annexin V antibody and propidium iodide (PI). Apoptotic cells were analyzed by flow cytometry. Result shown is the representative of results obtained in three independent experiments. **(B)** AGS cells were pre-treated with rJHP0290 (100 ng/ml) followed by addition of CPT (10 μM). Another set of cells were simultaneous treated with rJHP0290 (100 ng /ml) and CPT (10 μM). Percentage of apoptotic cells was determined. Values indicate mean ± SD of three independent experiments. **(C)** AGS, MKN45 and PHSEC cells were incubated either with protein storage buffer (Ctrl) or rJHP0290 (100 ng/ml) for 2 h followed by addition of corresponding volume of vehicle control or Camptothecin (CPT, 10 μM) for 14 h. Caspase 3 activity in the samples was determined as described in material and methods. Values indicate mean ± SD of three independent experiments. Statistically significant differences are indicated by * (p < 0.05).

The *H*. *pylori*-dependent regulation of apoptosis and proliferation in gastric epithelial cells has a major impact on ultimate disease outcome. *H*. *pylori* proteins, such as CagA and SlyD, have been shown to promote proliferation and exert an anti-apoptotic effect on gastric epithelial cells [15, 44]. Considering the induction of proliferation by rJHP0290, we further explored the rJHP0290-dependent regulation of apoptosis in AGS cells. CPT was used to induce apoptosis in AGS cells, and the induction of apoptosis was determined using FACS after staining with Annexin V and PI. A representative FACS analysis is shown in Fig 5A. At a concentration of 10 μM CPT, 26±3.5% of the cells were in early apoptosis (i.e., Annexin^+^ and PI^-^). Pretreatment of AGS cells with rJHP0290 for 2 h significantly reduced CPT-induced apoptosis (Fig 5B). However, the anti-apoptotic effect of rJHP0290 was markedly reduced when rJHP0290 and CPT were added to cells simultaneously (Fig 5B). rJHP0290 alone had no effect on AGS cell apoptosis at concentrations up to 10 μg/ml (data not shown).

The activation of caspase 3 is an important marker of apoptotic cell death. We further studied the effect of rJHP0290 on CPT-induced caspase 3 activity in gastric epithelial cells using a fluorimetric Caspase 3/7-activation assay kit. Gastric epithelial cells were pre-treated with rJHP0290, followed by the addition of CPT. Significantly reduced caspase 3 activity was observed in the rJHP0290 pre-treated groups (Fig 5C). These results suggested that JHP0290 inhibits CPT-induced apoptosis in gastric epithelial cells via the inhibition of caspase 3 activity.

### rJHP0290 activates NF-κB in gastric epithelial cells

**Fig 6:**
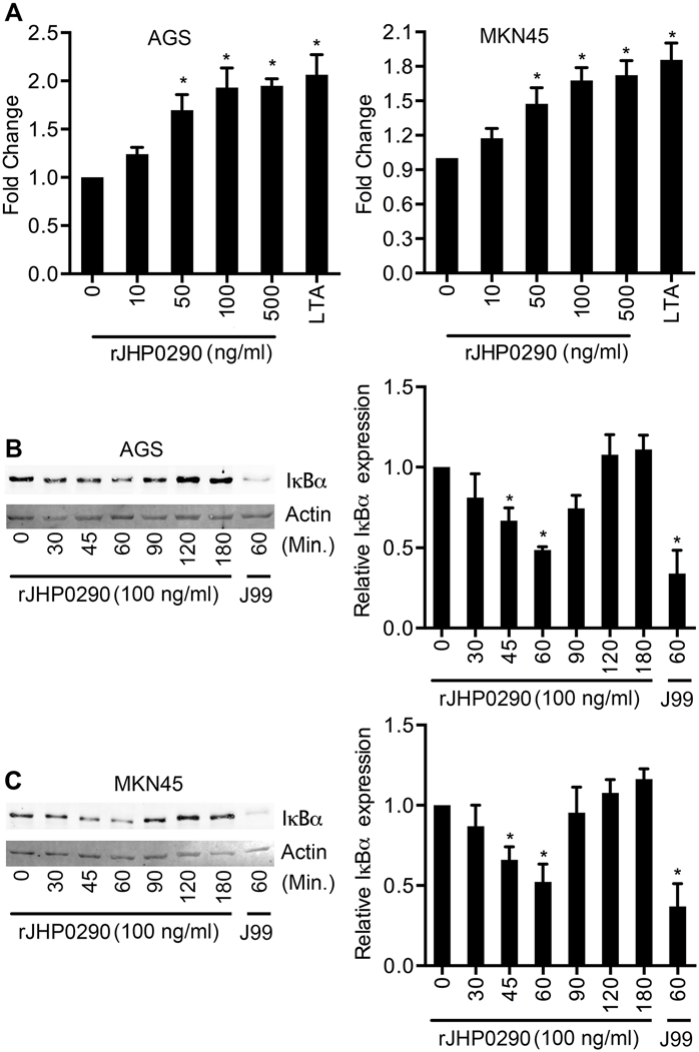
rJHP0290 activates NF-κB in AGS and MKN45 cells. **(A**) NF-κB-inducible SEAP reporter transfected AGS and MKN45 cells were treated with various concentrations of rJHP0290 as indicated in figure legends and activation of NF-κB was assessed using Quanti-Blue as substrate. Lipoteichoic acid (LTA) was used as positive control. Values indicate mean ± SD of three independent experiments. Statistically significant differences are indicated by * (p < 0.05). AGS **(B)** and MKN45 **(C)** cells were treated with rJHP0290 (100 ng/ml) for various time points (min) as indicated in figure legends. Cell lysates were prepared and immunoblotted with IκBα antibody followed by reprobing with anti-actin antibody to confirm equal loading. Blot shown is representative of results obtained in three independent experiments. The graph shows the densitometric analysis of western blot band intensities normalized to the actin control in three experiments. Statistically significant differences are indicated by * (p < 0.05).

NF-κB is a key transcription factor, which is involved in the regulation of various cellular responses against *H*. *pylori* [36]. We further assessed the effect of rJHP0290 on NF-κB using two approaches. AGS and MKN45 cells were transfected with the pNiFty-SEAP reporter plasmid, which is composed of three key elements: an ELAM proximal promoter, five NF-κB repeated transcription factor binding sites (TFBS) and a secreted alkaline phosphatase (SEAP) reporter gene. The cells were then treated with rJHP0290. SEAP activity in the culture supernatant was detected using the QUANTI-Blue detection system (InvivoGen). As shown in Fig 6A, rJHP0290 significantly induced SEAP activity in AGS and MKN45 cells in a dose-dependent manner. Using another approach, we determined the levels of IκBα in AGS and MKN45 cells after treatment with JHP0290. Treatment with rJHP0290 reduced the level of IκBα (Fig 6B and 6C), indicating that NF-κB was activated. IκBα began degrading 30 minutes post-treatment, and the basal IκBα level was restored after 90–120 min.

### rJHP0290 activates the ERK MAPK signaling pathway in gastric epithelial cells

**Fig 7:**
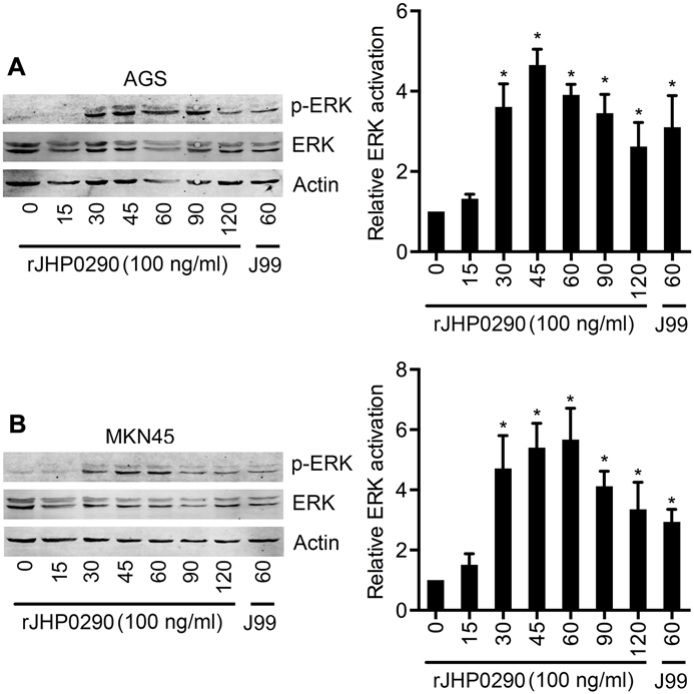
rJHP0290 activates ERK MAPK in gastric epithelial cells. AGS **(A)** and MKN45 **(B)** cells were treated with rJHP0290 for various time points (min) as indicated in the figure legends. Cell lysates were prepared and immunoblotted with anti-phospho-ERK antibody followed by reprobing with anti-ERK and anti-actin antibody to confirm equal loading. Blot shown is representative of results obtained in five independent experiments. The graph shows the western blot band intensities normalized to the actin control in five experiments. Statistically significant differences are indicated by * (p < 0.05).

ERK signaling pathways have been demonstrated to regulate *H*. *pylori*-induced proliferation in gastric epithelial cells [23, 31]. Therefore, we studied the activation of ERK after rJHP0290 challenge in both the AGS and MKN45 cell lines. Our results indicated that rJHP0290 induced the activation of ERK in a time-dependent manner (Fig 7A and 7B). The ERK activation kinetics was different in the two cell lines. In AGS cells, ERK activation could be detected as early as 30 min and peaked at 45 min, followed by a gradual reduction over a period of 120 min (Fig 7A). However, in the case of MKN45 cells, maximum activation was detected at 60 min (Fig 7B). A densitometry analysis of ERK phosphorylation kinetics from five independent experiments is shown in Fig 7A and 7B.

## Discussion

The balance between cell proliferation and cell death is critical for the maintenance of the integrity of the human gastric mucosa. *H*. *pylori* infection-associated disease development occurs as a result of the regulation of both proliferation and cell death by the bacterium and bacterium-derived products. Increased proliferation of gastric epithelial cells is a risk factor for gastric cancer. Although the whole bacterium and culture filtrates have been demonstrated to induce gastric epithelial cell proliferation [8, 13, 14, 16, 17, 31], only a small number of specific proliferation-promoting virulence factors, such as SlyD, *H*. *pylori* LPS, CagA and HspB, have been identified to date [15, 23, 29]. In this study, we identified JHP0290 as a novel regulator of gastric epithelial cell proliferation and apoptosis.

Sequence homology analysis indicated that JHP0290 homologues are highly conserved among *H*. *pylori* strains. The expression and release of the protein into the culture broth were detected for all strains tested in this study. However, it was interesting to note that the expression of JHP0290 homologues varied significantly among *H*. *pylori* strains. In addition, differences in the level of released protein were also observed; these differences could not be explained by differences in the expression level of the proteins among *H*. *pylori* strains. We are currently studying the expression and release of the protein in several additional strains, including clinical isolates from patients with various *H*. *pylori* infection-associated ailments. A similar discrepancy between the expression and release of the protein into the culture broth has been observed (Tavares, R et al., unpublished observations). The observed differences among the strains could be an intrinsic property of the strain or may occur due to differences in the environmental conditions (i.e., stress, pH, nutrients, level of mucins, etc.) among the sites of origin of the isolates. High genetic diversity exists among *H*. *pylori* isolates, and the final disease outcome is believed to be at least partially dependent on strain-specific factors. Strain-specific variations in the expression or release of several virulence-associated factors of *H*. *pylori* have been reported previously [45–47]. Further studies are required to understand the mechanism that underlies the observed differences in the expression and release of the JHP0290 homologues and the relationship, if any, between the concentration of this protein and the virulence of the bacterium.

rJHP0290 was found to exist primarily in the monomeric form, with a small percentage of the protein in the dimeric form. A similar pattern was observed for the endogenous JHP0290 protein in *H*. *pylori* strain J99. Considering the stronger binding of the dimeric form of the protein to epithelial cells and the significantly higher proliferation-promoting ability of the dimeric form, we speculate that differences in the levels of the dimeric and monomeric forms among various *H*. *pylori* strains may also affect ultimate host cell responses. We have observed differences in the levels of the monomeric and dimeric forms of the protein among *H*. *pylori* strains (Tavares, R et al., unpublished observations). We are currently unable to identify the factors that regulate the stability of the protein in the dimeric or monomeric form; thus, further studies are required.

Using an MTT assay and a BrdU Assay, we found that rJHP0290 induces proliferation in gastric epithelial cells. Endotoxin contamination is often observed in proteins purified from *E*. *coli*. Therefore, extreme care was taken to rule out the possibility that the observed effect was due to endotoxin contamination, as described in our previous study [42]. In addition, AGS and MKN45 cells naturally lack the MD2 protein, which is critical for the recognition of *E*. *coli* LPS [48]. We observed that an irrelevant His-tagged protein or boiled rJHP0290 failed to induce proliferation in gastric epithelial cells, further confirming that rJHP0290-specific effects occurred in the current experimental system. Furthermore, an anti-apoptotic effect of rJHP0290 on gastric epithelial cells was observed. This effect was regulated via the inhibition of caspase 3 enzymatic activity. Previous studies indicated that the anti-apoptotic effects of *H*. *pylori* or bacterium-derived products could occur due to the upregulation of anti-apoptotic genes, such as Mcl-1 or c-IAP2, or via the reduction of the level of caspase 3, as observed for the SlyD protein [15, 19, 49]. The possible rJHP0290-dependent regulation of anti-apoptotic genes or caspase 3 expression in gastric epithelial cells is under investigation. Furthermore, rJHP0290 affected AGS cell growth by inducing a faster progression into the cell cycle, as revealed by a higher percentage of cells in the S-G2-M phase of the cell cycle. Considering the proliferative and anti-apoptotic effects of JHP0290, the protein may act as a growth factor for epithelial cells.

It was intriguing to observe that rJHP0290 has a proapoptotic effect on macrophages [42] but an anti-apoptotic effect on gastric epithelial cells. We believe that the proapoptotic effect of rJHP0290 on macrophages is an indirect effect, which occurs primarily via the induction of TNF, which acts as a proapoptotic factor for macrophages [42]. In addition, the concentration of the protein also appears to determine the fate of different cell types. rJHP0290 was found to significantly induce apoptosis in RAW264.7 macrophages only at concentrations of 2 μg/ml and above. However, apoptosis was not observed in AGS and MKN45 cell lines at concentrations up to 10 μg/ml. For proliferation in AGS and MKN45 cells, a concentration of 10–1,000 ng/ml of the protein was sufficient and the proliferation-promoting property was lost at concentrations above 2 μg/ml.

MAPKs are important regulators of cellular events during *H*. *pylori* infection. rJHP0290 was found to activate ERK MAPK in a dose-dependent manner in both AGS and MKN45 cells. The role of ERK MAPK during *H*. *pylori*-induced gastric epithelial cell proliferation is well established [23, 31]. Furthermore, ERK MAPK has also been demonstrated to regulate the expression of the anti-apoptotic protein Mcl-1 in *H*. *pylori*-challenged gastric pits [19]. In addition to ERK MAPK, the Akt and Wnt signaling pathways are also known to regulate *H*. *pylori-*induced gastric epithelial cell proliferation [20]. Our preliminary results indicated that rJHP0290 induces the activation of Akt in AGS cells, as detected by the phosphorylation of Akt at Serine 473 (Tavares, R et al., unpublished observation). Therefore, rJHP0290 appears to regulate gastric epithelial cell proliferation and anti-apoptotic pathways via the ERK and Akt signaling pathways. A detailed analysis of the role of the above signaling pathways is currently being performed.

Activation of the key transcription factor NF-κB was observed in rJHP0290-challenged gastric epithelial cells. NF-κB is involved in the regulation of multiple cellular processes in cancer, including inflammation, proliferation and apoptosis [34]. NF-κB is a key regulator of pro-inflammatory cytokines and anti-apoptotic pathways during *H*. *pylori* infection [50]. The anti-apoptotic effects of *H*. *pylori* on gastric epithelial cells depend on the NF-κB-dependent regulation of the anti-apoptotic gene c-IAP2 [22]. Therefore, by regulating NF-κB activation, JHP0290 could potentially contribute to multiple events that are involved in the development of *H*. *pylori* infection-associated diseases. Various virulence factors from *H*. *pylori* are reported to induce the activation of NF-κB, including TIPα, HP0986, LPS, peptidoglycan and CagA [45, 50]. Although several studies suggested that cag pathogenicity island (*cag*PAI) is required for the optimal activation of NF-κB in gastric epithelial cells, other studies also demonstrated that purified proteins, such as HP0986 and TIP alpha, could also activate NF-κB in epithelial cells [45, 47]. The secretion of TIPα is not affected in a *cag*PAI mutant strain, and no currently available evidence supports the requirement for functional *cag*PAI in the release of HP0986 [45, 47]. In our preliminary studies, we failed to detect significant differences in the expression or release of the JHP0290 homologue in *H*. *pylori* strains 67:21 (*cag*PAI-positive) and 67:20 (*cag*PAI-negative).

In conclusion, this study provides evidence that suggests that JHP0290 may contribute to the development of *H*. *pylori* infection-associated diseases by promoting gastric epithelial cell proliferation, increased resistance to apoptosis and the activation of associated signaling pathways.

## Supporting Information

S1 Fig The expression of AhpC in different *H*. *pylori* strains. Whole cell lysate from equal number of cells of various *H*. *pylori* strains as indicated in figure legends were immunoblotted with anti-AhpC antibody. Blot shown is representative of results obtained in five independent experiments. The graph shows western blot band intensities quantified by the ImageJ software.
(TIF)

S2 Fig Monomeric and dimeric forms of rJHP0290. **(A)** rJHP0290 Wt and rJHP0290 C162A in the presence (+) or absence (-) of DTT in the SDS-PAGE sample buffer were analysed by SDS-PAGE followed by Coomassie blue staining. **(B)** Whole cell lysate (WCL) and culture broth (Sup) of *H*. *pylori* J99 Wt and J99 *Δjhp0290* was immunoblotted with anti-JHP0290 antibody. Blot shown is representative of results obtained in five independent experiments. A nonspecific band detected by the anti-JHP0290 antibody is marked by an arrow.
(TIF)
