# Supplementary figures and images for: Helicobacter pylori Protein JHP0290 Exhibits Proliferative and Anti-Apoptotic Effects in Gastric Epithelial Cells
Source: PLoS One. 2015 Apr 16;10(4):e0124407. doi: 10.1371/journal.pone.0124407 (PMC4400171; doi:10.1371/journal.pone.0124407)

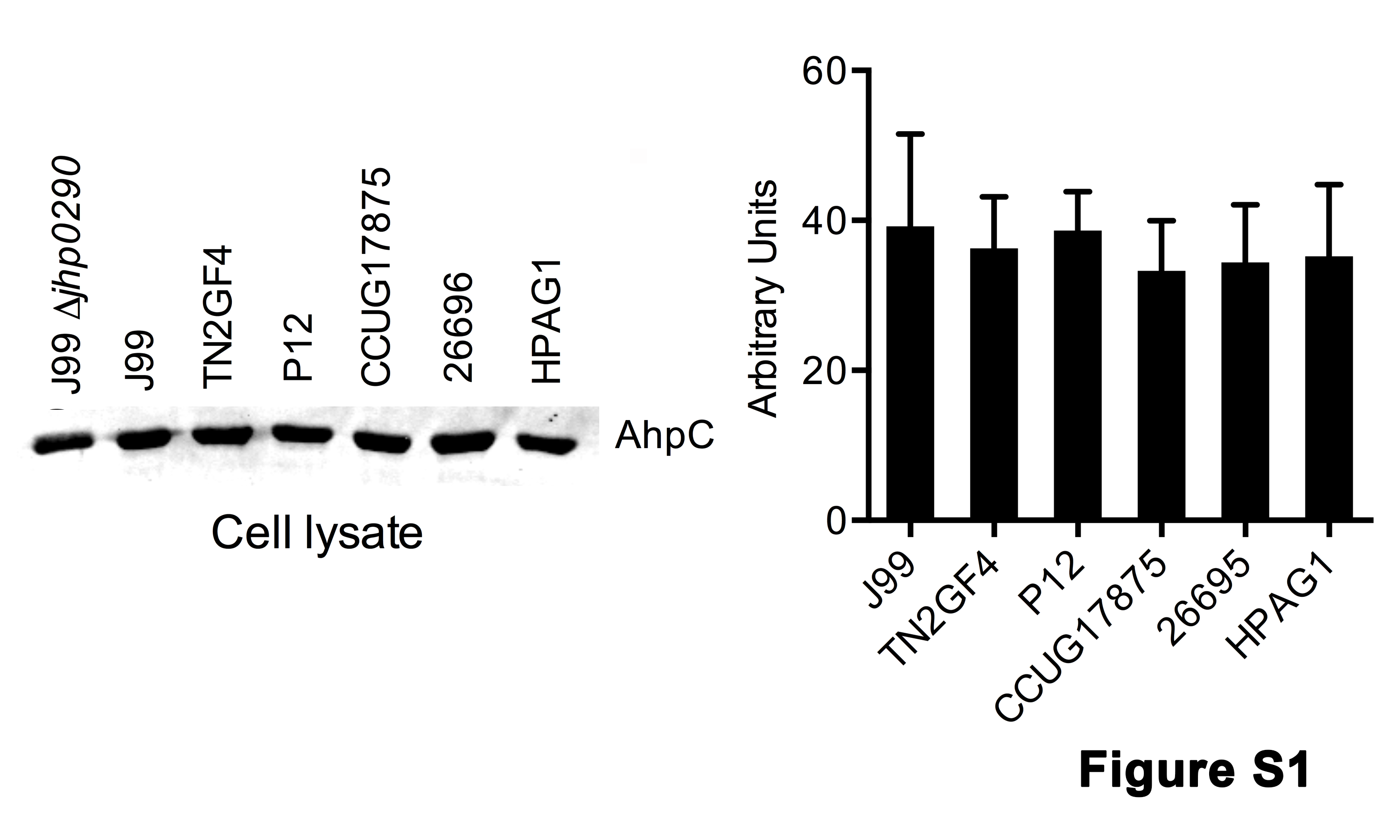

Supplement: S1 Fig — Whole cell lysate from equal number of cells of various H. pylori strains as indicated in figure legends were immunoblotted with anti-AhpC antibody. Blot shown is representative of results obtained in five independent experiments. The graph shows western blot band intensities quantified by the ImageJ software. (TIF) [file pone.0124407.s001.tif]

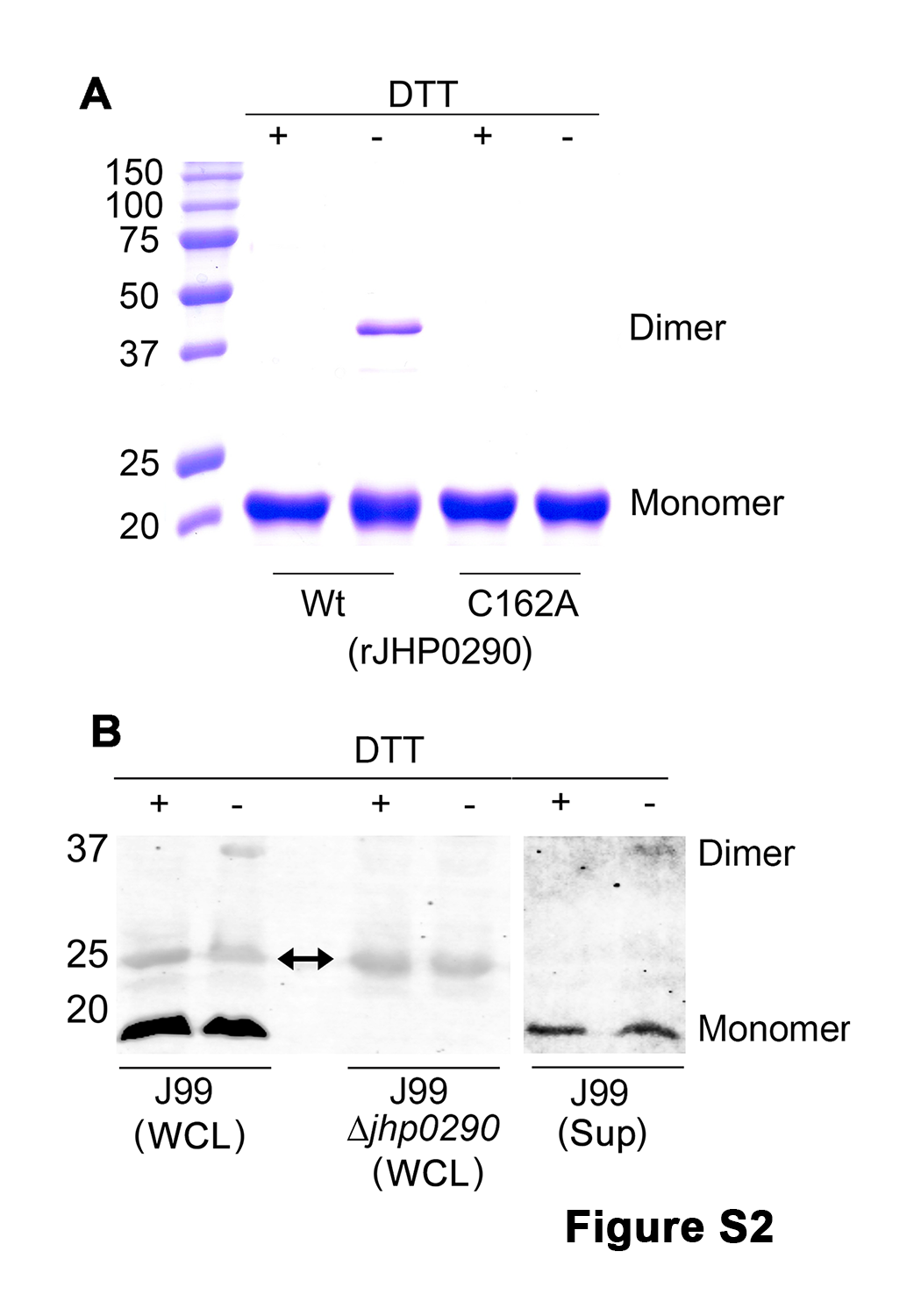

Supplement: S2 Fig — (A) rJHP0290 Wt and rJHP0290 C162A in the presence (+) or absence (-) of DTT in the SDS-PAGE sample buffer were analysed by SDS-PAGE followed by Coomassie blue staining. (B) Whole cell lysate (WCL) and culture broth (Sup) of H. pylori J99 Wt and J99 Δjhp0290 was immunoblotted with anti-JHP0290 antibody. Blot shown is representative of results obtained in five independent experiments. A nonspecific band detected by the anti-JHP0290 antibody is marked by an arrow. (TIF) [file pone.0124407.s002.tif]
